# The 5-CNL Front-of-Pack Nutrition Label Appears an Effective Tool to Achieve Food Substitutions towards Healthier Diets across Dietary Profiles

**DOI:** 10.1371/journal.pone.0157545

**Published:** 2016-06-20

**Authors:** Chantal Julia, Caroline Méjean, Sandrine Péneau, Camille Buscail, Benjamin Alles, Léopold Fézeu, Mathilde Touvier, Serge Hercberg, Emmanuelle Kesse-Guyot

**Affiliations:** 1 Université Paris 13, Equipe de Recherche en Epidémiologie Nutritionnelle (EREN), Centre d'Epidemiologie et Biostatistiques Sorbonne Paris Cité (CRESS), Inserm U1153, Inra U1125, Cnam, COMUE Sorbonne-Paris-Cité, F-93017, Bobigny, France; 2 Département de Santé Publique, Hôpital Avicenne (AP-HP), F-93017, Bobigny, France; TNO, NETHERLANDS

## Abstract

**Background:**

Front-of-pack (FOP) nutrition labels are considered helpful tools to help consumers making healthier food choices, thus improving their diet. In France, the implementation of a FOP nutrition label–the 5-Colour Nutrition Label (5-CNL)–is currently under consideration. Our objective was to investigate dietary profiles in a French adult population using the 5-CNL, and to assess its potential impact in improving the diet through substitution of foods.

**Methods and Findings:**

Subjects included in the NutriNet-Santé cohort, who had completed three 24-h dietary records were included in this cross-sectional analysis. Mutually exclusive clusters of individuals were identified using the percentage of energy derived from foods of each of the 5-CNL colours as input variables. Three scenarios of substitution of foods for healthier alternative using the 5-CNL were tested. Food group and dietary intakes, socio-demographic and lifestyle data were compared across clusters using ANOVAs or Chi-square tests, as appropriate. We identified three mutually exclusive dietary profiles: ‘Healthy’ (N = 28 095, 29.3% of the sample), with high consumption of fruit, vegetables, whole cereals and fish; ‘Western’ (N = 33 386, 34.8%); with high consumption of sweetened beverages, breakfast cereal, cheese, fatty and sugary foods; ‘Traditional’ (N = 34 461, 35.1%), with high consumption of potatoes, bread, meat and dairy desserts. Overall, substitutions strategies led to an increase in the number of subjects reaching the recommended intakes in energy, macro and micronutrients. Increases were particularly high in the ‘Western’ pattern for lipids and saturates intakes: from 16.2% reaching the recommended amount for lipids (13.5% for saturates) to 60.6% and 85.7% respectively.

**Conclusion:**

The use of the 5-CNL as an indicator of food choice meaningfully characterizes clusters of dietary habits and appears as an effective tool to help improving the nutritional quality of the diet.

## What This Paper Adds

### What Is Already Known on the Subject

- In France, the introduction of the 5-Colour Front-of-pack Nutrition Label (5-CNL) is under consideration. The 5-CNL is based on a nutrient profiling system and classifies foods into 5 categories of nutritional quality, from ‘Green’ to ‘Red’.
- The 5-CNL has been shown to adequately classify foods according to their nutritional quality, and to help consumers understanding nutritional quality of foods and making healthier food choices.
- The contribution of each of the 5-CNL ‘colour’ foods in individual diets, as well as the potential impact of food substitutions using the 5-CNL on nutritional quality of the diet has not been investigated.

### What This Study Adds

- Contribution of each ‘colour’ of the 5-CNL allowed for the identification of three dietary profiles:‘Healthy’, ‘Western’ and ‘Traditional’
- The use of the 5-CNL for food substitutions strategies led to an increase in the number of subjects reaching the recommended amounts in macro and micro-nutrients, with a more important impact for subjects with lower nutritional quality profiles, highlighting the potential contribution of the 5-CNL to public health nutrition programs.

## Introduction

**Fig 1:**
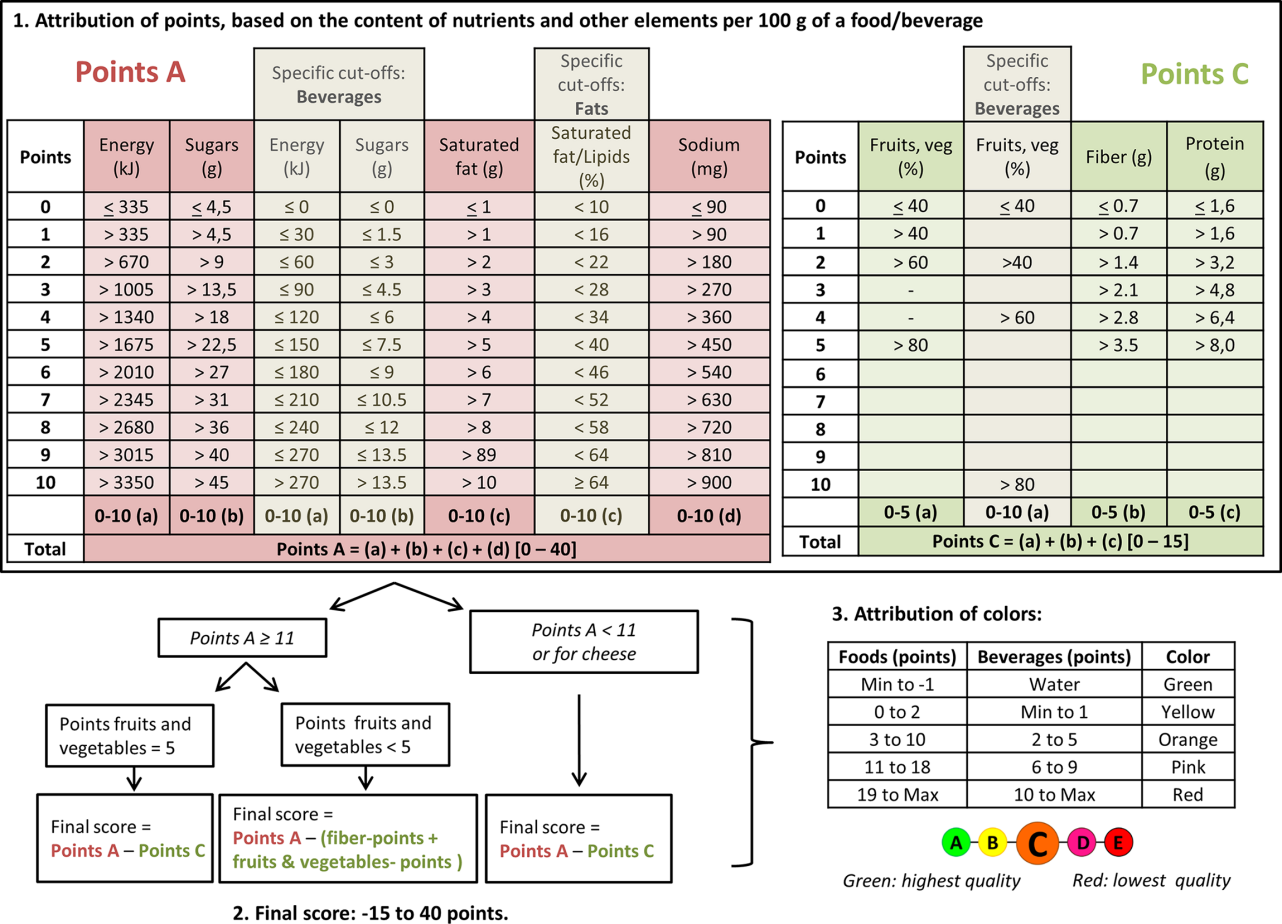
FSA score computation and 5-CNL allocation. Footnotes: Exceptions were made for cheese, fat, and drinks to take into account their specific composition, consistently with nutritional recommendations. The percentage of fruits and vegetables was calculated taking into account fruits, legumes and vegetables as defined in the PNNS (the French nutritional and health policy). Tubers, oleaginous fruits, dried fruits and olives are therefore not considered in this computation. FSA score allocates different thresholds for fibers, depending on the measurement method used. We used NSP cut-offs to compute fibers score.

Front-of-pack (FOP) nutrition labels have received growing attention from policy makers worldwide, as they are considered promising tools in public health nutrition [1,2]. The introduction of a FOP nutrition label is thought to help consumers making healthier choices in purchasing situations [2,3]. Moreover, it is believed that they would serve as incentives for manufacturers to reformulate their products towards healthier compositions [2,3]. Some western countries have introduced FOP nutrition labels, the earliest adopters being Sweden in 1989 (*Green Keyhole*) [4], and more recently the Netherlands in 2006 (*Choices*) [5], or Australia and New Zealand in 2014 (*Health Star Rating System*) [6]. In France, the government has recently proposed to introduce a simplified FOP nutrition label [7] following a report to the Minister of Health in 2014 [8]. This label would complement the set of measures coordinated by the French Nutrition and Health Program (Programme National Nutrition Santé, PNNS), a nationwide program initiated in 2001 and aiming at improving health in the population by acting on one of its main modifiable determinants, nutrition [9]. The PNNS actions include population education and information through multimedia communication campaigns and dissemination of booklets, local initiatives of health promotion and modification of the environment (e.g. modification of the walkability of the built environment) and state-level regulations [9]. The proposed label is based on the British Food Standards Agency nutrient profiling system (FSA score) currently used to regulate advertising of foods and beverages to children in the United Kingdom (UK) [10,11]. This nutrient profiling system provides a global assessment of the nutritional quality of foods and beverages, combining into one indicator several nutrients entering the composition of the food or beverage. Among the various nutrient profiling systems currently available, the FSA score has been estimated as one of the most validated [12,13], and is currently serving as a basis for several public health initiatives, including the Australian *Health Star Rating System* [6]. The French proposal derives five categories of nutritional quality from the continuous FSA score, each associated with a different colour, from ‘Green’ for the highest nutritional quality category to ‘Red’ for the lowest nutritional quality category, representing the 5-Colour Nutrition Label, (5-CNL) (see **Fig 1**) [8].

This approach, using a global indicator of nutritional quality, with an ‘across-the-board’ application (i.e. the score computation is identical whatever the food group considered) has been found helpful to guide consumers’ food choices toward food groups with higher nutritional quality instead of low nutritional quality groups (‘displacement’). It also allows consumer to choose products with higher nutritional quality among the products from the same food group (‘substitution’).[14].

The application of the FSA score to foods in the French context indicated that the score could be used as a basis for a five-category system, provided marginal adjustments in a few food groups (beverages, cheese and fats) were made to maintain a high consistency with the French official nutritional recommendations [15]. Moreover, the use of the 5-CNL was shown to accurately discriminate the nutritional quality between food groups, within a food group and for similar products from different brands [16,17]. For example, vegetables and whole grains are mainly classified as ‘Green’ while chocolate products are mainly classified as ‘Red’. Moreover, in a given food group, for example breakfast cereals, oat meals are classified as ‘Green’ while chocolate stuffed cereals are classified as ‘Red’[17]. These elements confirms the potential of the 5-CNL for the implementation of both ‘displacement’ and ‘substitution’ strategies in individual diets.

However, the potential impact of the use of the 5-CNL label on individual diets has not been investigated. The heterogeneity in dietary behaviour observed in the population suggests that the use of the label, and therefore its impact, would also be heterogeneous. Identifying dietary profiles using the 5-CNL classification would allow investigating the potential impact of the 5-CNL across various dietary profiles.

Our objectives were therefore: 1) to identify dietary profiles according to the 5-CNL classification of the foods consumed; 2) to investigate the potential impact of the substitution of foods using the 5-CNL across dietary profiles through simulations. The highest possible gain in nutritional quality of the diet through the substitution of all foods was simulated, as well as a scenario involving the substitution of a fraction of the foods consumed. The overall aim was to assess the potential gain in the nutritional quality of the diet at the population level through this novel tool, in a public health perspective.

## Material and Methods

### Population

Participants were selected from the NutriNet-Santé cohort. Briefly, the NutriNet-santé study is a prospective cohort study set in France in which inclusion and follow-up of volunteer participants are performed on the Internet [18]. The main objectives of the NutriNet-santé study are: 1) to investigate the relationship between nutrition and health outcomes; and 2) to investigate the determinants of dietary patterns and nutritional status. Inclusion in the study began in May, 2009, and is still ongoing. Volunteer participants aged >18 years-old subscribe to the study, and are included when they have completed a set of questionnaires assessing: diet (through repeated 24h dietary records), physical activity, anthropometry, lifestyle and socio-economic conditions and health status. Detailed information on the NutriNet-Santé study can be found elsewhere [18].

For the present study, all participants having completed three dietary records at baseline, with no under-reporting of energy intake [19] were included.

### Ethics

The NutriNet-Santé study is conducted in accordance with the Declaration of Helsinki, and all procedures have been approved by the Institutional Review Board of the French Institute for Health and Medical Research (0000388FWA00005831) and the *Commission Nationale de l’Informatique et des Libertés* (908450 and 909216). Electronic informed consent was obtained from all participants. The Nutrinet-santé study is registered under EudraCT registration number 2013-000929-31.

### Socio-Demographic and Lifestyle Data

Socio-demographic and lifestyle data were collected at enrollment through self-administered questionnaires, including age, gender, education (no diploma and primary education, secondary education, university, other), marital status (in couple, single/divorced/widowed), income per household unit [20] (<900, 900–1199, 1200–1799, 1800–2299, 2300–2699, 2700–3699, >3700 €/month) and smoking status (current smoker, former smoker and never smoker). Physical activity was computed using self-declared data from the International Physical Activity Questionnaire, completed at baseline (low, moderate and high physical activity levels) [21]. Self-reported weight and height were collected at baseline and were used to compute body mass index (WHO categories: <18.5, [18.5–25[, [25–30[, ≥30kg/m^2^) [22].

### Dietary Data

Dietary data were derived from three repeated 24-hour records at enrollment, randomly distributed in a two-week period, with two week days and one week-end day. Food consumption was weighted according to the representativeness of the day of the week of each record in the whole diet (five days for a week day and two days for week-end days). Portion size for each reported food and beverage item was estimated by participants using validated photographs [23]. Nutrient intake was computed using a published food composition database structured for epidemiologic purposes and including 70 dietary compounds reflecting foods usually consumed in the French diet [24]. No information on brand or price of foods was included in the records. Under- reporters for energy intake were identified using Goldberg/Black’s method and were excluded [19].

### Score Computation, 5-CNL Allocation and Application to the Diet

#### Score computation

The FSA score for foods and beverages was computed taking into account the nutrient content for 100g [11]. It allocates positive points (0–10) for content in energy (KJ), total sugar (g), saturated fatty acids (g) and sodium (mg). Negative points (0–5) are allocated to content in fruits, vegetables, legumes and nuts (%), fibers (g) and proteins (g). Scores for foods and beverages were therefore based on a discrete continuous scale from -15 (most healthy) to +40 (less healthy) **(Fig 1).** Modifications to the original score were used in order to ensure a higher consistency with French nutritional recommendations for dried fruits and nuts, beverages, cheese and added fats [25].

#### 5-CNL allocation

Products were then classified into five categories, corresponding to the 5-CNL classification. The cut-offs to define the five categories of the 5-CNL were based on the independent reports conducted by the French Agency for Food, Environmental and Occupational Health Safety (ANSES) and the High Council for Public Health (HCSP) [25,26], as follows from higher nutritional quality to lower nutritional quality: for foods ‘Green’ (-15 to -1), ‘Yellow’ (0 to 2), ‘Orange’ (3 to 10), ‘Pink’ (11 to 18) and ‘Red’ (19 and above); for beverages ‘Green’ only for non-flavoured water and non-sugared hot beverages, ‘Yellow’ (Min to 1), ‘Orange’ (2 to 5), ‘Pink’ (6 to 9) and ‘Red’ (10 and above) (**Fig 1**).

#### Dietary clusters

For each individual, the mean daily energy intake (excluding energy from alcoholic beverages) derived from the foods of each colour of the 5-CNL was computed. The mean daily energy intake of each individual was therefore separated in ‘Energy from Green-labelled foods’ to ‘energy from Red-labelled foods’. The five variables obtained were used as input variables in a clustering procedure (SAS CLUSTER and FASCLUS procedures). The plot of the semi-partial R^2^, the semi-partial T^2^ and the Cubic Clustering Criterion by the number of clusters were used to identify that a model with three mutually exclusive clusters of individuals was adequate.

### Scenarios of Substitution

In order to investigate the potential impact of substitutions of foods using the 5-CNL, we modelled substitutions within food groups of similar use, using a gradual approach. Three scenarios of substitution were modelled, in order to highlight the potential gain in diet quality through substitution (**S1 Fig**). Food groups tested for the substitution are presented in **S1 Table**.

#### Simulation scenario n°1 substitution of all food for all healthier foods

In this first scenario, foods classified in the highest nutritional quality class of the 5-CNL within a food group were considered not substituted. Foods and beverages classified in lower nutritional quality classes of the 5-CNL were substituted, and were attributed the mean nutritional values of all the healthier foods and beverages (e.g. Red-labelled foods were attributed the mean nutritional values of foods from the ‘Pink’ to the ‘Green’ classes). The initial quantity of the food was retained. This scenario models the substitution from lower nutritional quality foods, as classified in the 5-CNL to higher nutritional quality foods, with an even probability of choice for all foods in healthier classes of the 5-CNL. This scenario was used to investigate the highest nutritional gain possible through substitutions.

#### Simulation scenario n°2 substitution of all food for healthier foods in the adjacent class of the 5-CNL

This second scenario is similar to the previous, but substituted foods were attributed the mean nutritional values of foods in the immediately adjacent colour within a food group (e.g. Red-labelled foods were attributed the mean nutritional values of foods in the ‘Pink’ category).

#### Simulation scenario n°3 Scenario n°2 applied to 30% of foods

In this third scenario, 30% of foods were randomly selected in each 24h-record for each subject, and were applied the second scenario of substitution. This scenario, retaining a fraction of eligible foods for substitution was considered as the most realistic in real-life settings.

### Statistical Analysis

Sociodemographic, lifestyle, anthropometrics, food group consumption and nutrient intake were compared across clusters using ANOVAs or Chi-square tests, as appropriate. Energy-adjusted nutrient intakes [27] were compared across clusters. Percentages of subjects with adequate intakes (energy, lipids, carbohydrates, proteins, added sugars, saturates, fibers) according to French national recommendations were compared across clusters and scenarios of substitution. Cut-offs for adequate intakes were determined using French national nutrition and health program objectives (Programme National Nutrition Santé, PNNS)[28] or recommendations from the French Agency for Food, Environmental and Occupational Health Safety (ANSES)[29] (**See S2 Table**). Basal metabolic rate and individual expenditure were computed using Schofield equations. Energy intakes <105% of expenditures were considered as adequate [30].

All tests were two-sided and a P value <0.001 was considered significant, given the high number of statistical tests performed. Statistical analyses were performed using SAS Software (version 9.3, SAS Institute Inc, Cary, NC, USA). Figures were obtained using R software (version 3.0.3)

## Results

Among the 158 291 subjects included in the NutriNet-Santé study up to March 23^rd^, 2015, 95 942 subjects had three available normo-energy dietary records at baseline and were included in the analysis. The sample included 78.1% of women, and the mean age of the sample was 43.1 ± 14.6 years old.

The clustering procedure identified three mutually exclusive groups of subjects (**Table 1**). In all clusters, the proportion of energy from foods from each colour category was >8% of total energy intake. Clusters were characterized and labelled according to the food group consumption patterns observed, as: ‘Healthy’ (N = 28 095, 29.3% of the sample), ‘Western’ (N = 33 386, 34.8%) and ‘Traditional’ (N = 34 461, 35.1%).

**Table 1:** Consumption of foods from each colour (% energy intake) and sociodemographic, lifestyle and anthropometric characteristics across identified clusters (N = 95942).

	Cluster 1 = HEALTHY	Cluster 2 = WESTERN	Cluster 3 = TRADITIONAL	P
N	28095	33386	34461	
Consumption from 'colour' foods				
Green	44 ± 9.81	21.2 ± 7.93	22.5 ± 7.29	<0.0001
Yellow	13.8 ± 7.63	14.6 ± 6.88	27.3 ± 9.05	<0.0001
Orange	13.2 ± 8.62	11.7 ± 7.51	13.1 ± 8.39	<0.0001
Pink	20.6 ± 8.22	40.3 ± 7.94	22.8 ± 6.94	<0.0001
Red	8.31 ± 6.84	12.2 ± 7.88	14.4 ± 9.16	<0.0001
Sex				
Male	5440 (19.4)	6866 (20.6)	8708 (25.3)	<0.0001
Female	22655 (80.6)	26520 (79.4)	25753 (74.7)	
Age category (y)				
18–24 years old	1965 (7)	5657 (16.9)	3569 (10.4)	<0.0001
25–44 years old	9315 (33.2)	16567 (49.6)	14329 (41.6)	
45–64 years old	14023 (49.9)	9895 (29.6)	14026 (40.7)	
≥ 65 years old	2792 (9.9)	1267 (3.8)	2537 (7.4)	
Monthly income (€)				
<900	2548 (9.1)	4172 (12.5)	3606 (10.5)	<0.0001
900–1200	1522 (5.4)	2321 (7)	2414 (7)	
1201–1800	6425 (22.9)	8462 (25.3)	8641 (25.1)	
1801–2300	4227 (15)	4712 (14.1)	4814 (14)	
2301–2700	2591 (9.2)	2753 (8.2)	3010 (8.7)	
2701–3700	4249 (15.1)	4135 (12.4)	4910 (14.2)	
>3700	3105 (11.1)	2765 (8.3)	3212 (9.3)	
Missing	3428 (12.2)	4066 (12.2)	3854 (11.2)	
Educational level				
No diploma and primary	952 (3.4)	701 (2.1)	1044 (3)	<0.0001
Secondary	9565 (34)	10429 (31.2)	12006 (34.8)	
University	17365 (61.8)	22049 (66)	21169 (61.4)	
Other	213 (0.8)	207 (0.6)	242 (0.7)	
Living area				
Urban	21691 (77.2)	25832 (77.4)	26456 (76.8)	<0.0001
Rural	5825 (20.7)	6892 (20.6)	7591 (22)	
Abroad	579 (2.1)	662 (2)	414 (1.2)	
Marital status				
Single/divorced/widowed	8497 (30.3)	10314 (30.9)	9031 (26.2)	<0.0001
In couple	19589 (69.7)	23065 (69.1)	25419 (73.8)	
Smoking statut				
Never smoker	14135 (50.3)	16989 (50.9)	16784 (48.7)	<0.0001
Former smoker	10650 (37.9)	9778 (29.3)	11779 (34.2)	
Current smoker	3301 (11.8)	6612 (19.8)	5891 (17.1)	
Body mass index category				
Underweight	1548 (5.5)	1971 (5.9)	1585 (4.6)	<0.0001
Normal weight	17558 (62.5)	22244 (66.6)	21689 (62.9)	
Overweight	6368 (22.7)	6416 (19.2)	7838 (22.7)	
Obese	2611 (9.3)	2751 (8.2)	3343 (9.7)	

Data are N(%) and Mean ± SD. P value for Chisquare tests across clusters

### Socio-Demographic Characteristics

Subjects in the ‘Traditional’ pattern were more often men, middle-aged, with low incomes, living in a rural setting, and were more likely overweight or obese (**Table 1**). Subjects in the ‘Western’ pattern were younger, with low income and higher education (university students) and were more often smokers (**Table 1**). Subjects in the ‘Healthy’ pattern were more likely women, middle-aged, with high income, living in urban areas and former smokers (**Table 1**).

### Food Consumption and Nutritional Intake

The ‘Healthy’ pattern was characterized by high consumption of fruit, vegetables, legumes, whole cereals and fish and seafood (**Table 2**). This led to high intakes in fibers, vitamins and minerals (**Table 3**). The ‘Western’ pattern was characterized by high consumption of sweetened beverages, breakfast cereal, cheese and fatty and sugary foods (**Table 2**). This led to high intakes in energy, saturated fat, cholesterol, and added sugar (**Table 3**). The ‘Traditional’ pattern was characterized by high consumption of potatoes, bread, meat and dairy desserts (**Table 2**). This led to high sodium and added fat intakes (**Table 3**).

**Table 2:** Food group consumption (g/day) according to the identified clusters (N = 95942).

	Cluster 1 = HEALTHY	Cluster 2 = WESTERN	Cluster 3 = TRADITIONAL	P
N	28095	33386	34461	
Fruits and vegetables	550 ± 264	335 ± 185	378 ± 191	<0.0001
Fruits	270 ± 188	149 ± 125	171 ± 131	<0.0001
Vegetables	280 ± 143	185 ± 106	206 ± 110	<0.0001
Starchy foods	254 ± 125	219 ± 93.5	261 ± 106	<0.0001
Pasta, rice and bread	120 ± 92.7	131 ± 75.9	174 ± 88.9	<0.0001
Whole grains	62.9 ± 66.1	26.6 ± 37.9	20.9 ± 33.1	<0.0001
Breakfast cereals	6.56 ± 16.5	9.12 ± 20.1	5.21 ± 14.4	<0.0001
Potatoes and tubers	46.3 ± 55	43.2 ± 47.6	51 ± 52.1	<0.0001
Legumes	18.1 ± 35.3	9.03 ± 21.9	10.3 ± 23.4	<0.0001
Fish, meat and eggs	162 ± 86.7	148 ± 71.5	165 ± 74.8	<0.0001
Meat and poultry	82.8 ± 62.7	78.6 ± 53.6	88 ± 57.3	<0.0001
Fish and seafood	51.1 ± 51.2	31.1 ± 37.5	35.6 ± 40.2	<0.0001
Processed meat and fish	15.2 ± 23.7	25.5 ± 30.2	26 ± 31.1	<0.0001
Eggs	13.2 ± 21.6	12.5 ± 19.7	15.2 ± 23.1	<0.0001
Milk, dairy and fresh desserts	261 ± 186	221 ± 148	232 ± 154	<0.0001
Dairy desserts	27.1 ± 47.5	36.6 ± 52.2	43.4 ± 59.7	<0.0001
Cheese	27.4 ± 25	44.8 ± 33.4	35.5 ± 27.7	<0.0001
Milk	90.3 ± 140	79.7 ± 119	80.5 ± 122	<0.0001
Yogurt and cottage cheese	116 ± 116	59.5 ± 70.9	72.8 ± 77.7	<0.0001
Fats	23 ± 15.9	26.7 ± 18.4	25.2 ± 16.5	<0.0001
Sugary products	62.9 ± 47.9	128 ± 75.4	88.2 ± 60.1	<0.0001
Dried fruits	3.37 ± 11.2	1.98 ± 8.22	1.8 ± 7.01	<0.0001
Biscuits and cakes	23.7 ± 31	56.6 ± 55.5	38.9 ± 43.8	<0.0001
Pastries	4.03 ± 11.4	17.6 ± 28	7.33 ± 15.3	<0.0001
Fatty and sugary products	12.9 ± 20.8	29.8 ± 37.9	18.5 ± 26.5	<0.0001
Confectionery	18.9 ± 21.9	22.4 ± 24.7	21.7 ± 22.4	<0.0001
Salty snacks	12.7 ± 19.8	19.8 ± 23	14.1 ± 18.8	<0.0001
Beverages	1340 ± 616	1320 ± 575	1280 ± 563	<0.0001
Non-sugared beverages	1200 ± 606	1080 ± 555	1070 ± 543	<0.0001
Soft drinks	20.9 ± 61.2	69 ± 135	48.1 ± 112	<0.0001
Fruit and vegetable juice	43.9 ± 76.1	70.8 ± 96.4	54.1 ± 82.4	<0.0001
Alcoholic beverages	76.2 ± 129	104 ± 165	107 ± 169	<0.0001

Data are Means±SD in g/day. P value for ANOVA across clusters

**Table 3:** Energy, macro- and microutrient intake in the identified clusters (N = 95942).

	Cluster 1 = HEALTHY	Cluster 2 = WESTERN	Cluster 3 = TRADITIONAL	
N	28095	33386	34461	P
Energy intake (kcal/d)	1740 ± 447	2000 ± 510	1930 ± 495	<0.0001
Carbohydrates (g/day)	196 ± 35.4	186 ± 34.5	194 ± 34.7	<0.0001
Lipids (g/day)	73.1 ± 13.5	83.3 ± 12.8	76.6 ± 13.2	<0.0001
Proteins (g/d)	84 ± 19	71.9 ± 14.1	76.7 ± 14.3	<0.0001
Sugars (g/d)	93.5 ± 26.8	91.8 ± 28.2	85.5 ± 26.4	<0.0001
Added sugars (g/d)	28.7 ± 16.4	44.3 ± 22.9	35.4 ± 20.6	<0.0001
Added fat (g/d)	22 ± 11	22 ± 12.3	23.4 ± 11.7	<0.0001
Added animal fat (g/d)	6.71 ± 6.29	7.44 ± 7.24	8.7 ± 7.85	<0.0001
Added vegetable fat (g/d)	15.3 ± 9.94	14.6 ± 10.9	14.7 ± 9.87	<0.0001
Saturated fat (g/d)	27.4 ± 7.19	35.6 ± 7.55	31.3 ± 7.43	<0.0001
Monounsaturated fat (g/d)	28.1 ± 7.49	31.1 ± 7.1	28.7 ± 6.74	<0.0001
Polyunsaturated fat (g/d)	11.9 ± 4.94	10.8 ± 4.23	10.8 ± 4.4	<0.0001
Cholesterol (g/d)	281 ± 128	311 ± 116	309 ± 123	<0.0001
Sodium (mg/d)	2600 ± 705	2500 ± 701	2720 ± 678	<0.0001
Fibres (g/d)	24.1 ± 7.46	16.5 ± 5.21	17.7 ± 5.11	<0.0001
Beta-carotene (μg/d)	4410 ± 3390	2870 ± 2350	3180 ± 2380	<0.0001
Calcium (mg/d)	978 ± 277	884 ± 267	863 ± 246	<0.0001
Iron (mg/d)	15.2 ± 4.41	11.9 ± 3.7	12.4 ± 3.82	<0.0001
Magnesium (mg/d)	396 ± 112	297 ± 82.3	303 ± 75.9	<0.0001
Vitamin B6 (mg/d)	2.02 ± 0.56	1.55 ± 0.49	1.62 ± 0.46	<0.0001
Vitamin B9 (μg/d)	384 ± 123	293 ± 93.8	304 ± 95.5	<0.0001
Vitamin B12 (μg/d)	5.77 ± 5.85	4.53 ± 4.2	5.03 ± 5.19	<0.0001
Vitamin C (mg/d)	138 ± 85.2	108 ± 86.1	108 ± 74.5	<0.0001

Data are Mean ±SD. Macro and Micronutrient intake adjusted on energy intake.

### Substitution Scenarios

**Fig 2:**
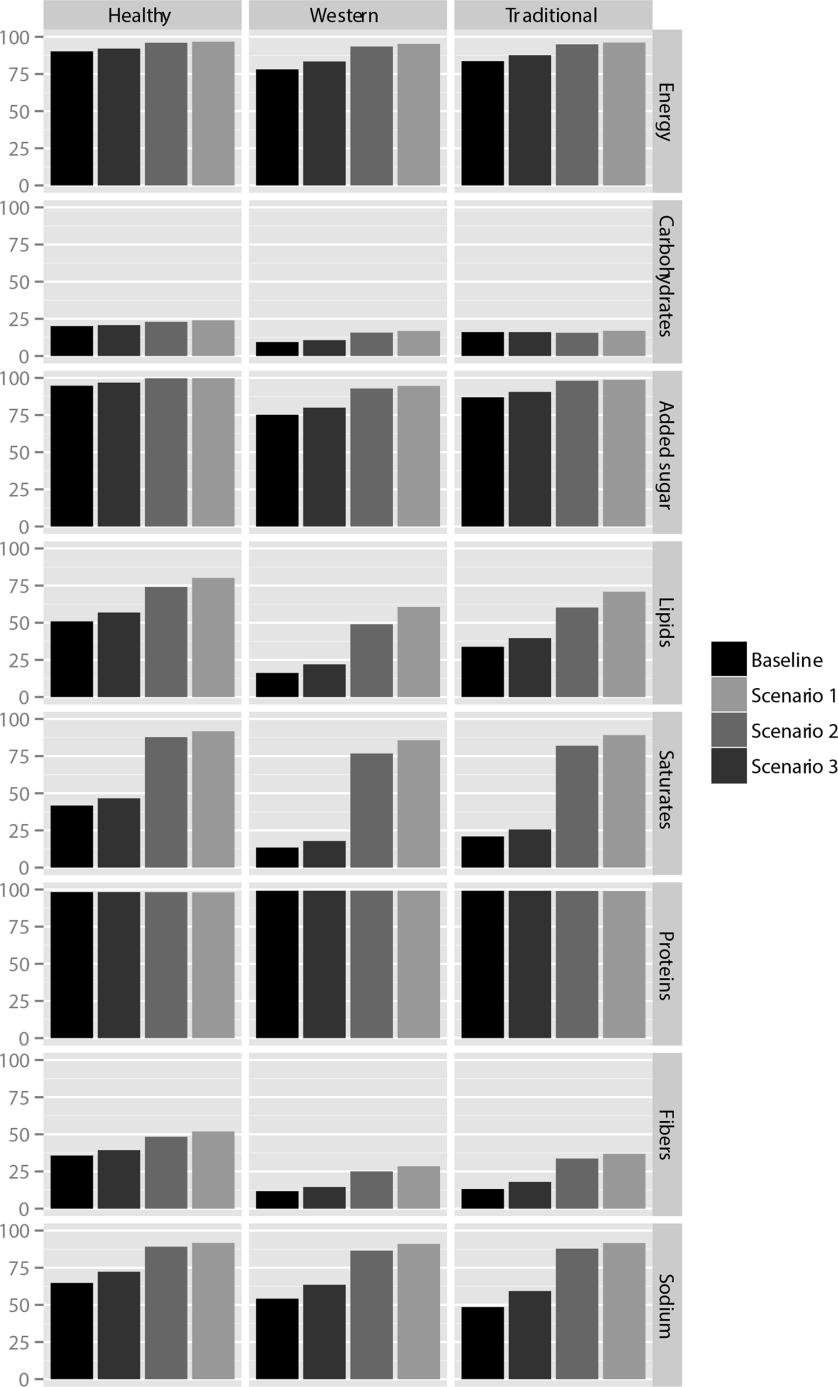
Percentage of subjects reaching the recommended amounts for energy, lipids, proteins, carbohydrates, added sugar, saturates and fibers across substitution scenarios and clusters.

Overall, substitutions strategies led to a decrease in lipid, sugars and added sugars intakes and an increase in fibers intakes, leading to an increase in the number of subjects reaching the recommended intakes in energy, macro and micronutrients (**Fig 2**). The most important increases were observed for lipids and saturates intakes, more particularly in the ‘Western’ patterns (**Fig 2**). Before substitution, 16.2% of subjects in the ‘Western’ pattern reached the recommended amount of lipid intakes (13.5% for saturates,); in scenario n°3, 22.0% reached the recommended amount for lipids (17.8% for saturates); finally, in scenario n°1, 60.6% reached the recommended amount for lipids (85.7% for saturates) (**Fig 2**). On the other hand, as expected, the number of subjects reaching the recommended intakes in the ‘Healthy’ pattern was more important at baseline, but the magnitude of effect of the substitution scenarios in this group of consumers was lower (e.g. 50.9% of subjects reached the recommended lipid amount before substitution, and they were 56.8% in scenario n°1) (**Fig 2**).

For added sugars, fibers and sodium, substitutions using the 5-CNL significantly increased the number of subjects reaching the recommended amounts, but the magnitude of these effects was comparatively lower. For carbohydrates and proteins intakes, substitutions using the 5-CNL did not modify significantly the number of subjects reaching the recommendations.

## Discussion

Our results allowed for the identification of three mutually exclusive dietary profiles using the 5-CNL, characterized by specific dietary behaviours. Whatever the cluster considered, foods from each colour category of the 5-CNL composed a substantial part of the diet. Finally, the simulated substitution of foods allowed for substantial modifications in macro- and micro-nutrient intakes in individuals, with a higher impact in patterns characterized with a lower diet quality. Though scenario n°1 appears unrealistic, it shows the margin of progression attainable in the population, highlighting the potential of the 5-CNL to contribute in reaching the French National Nutrition and Health Program (PNNS) objectives.

The dietary profiles that were identified in our study are consistent with the existing literature [31–33]. Subjects with healthier dietary profiles have been frequently reported to be more often women, with higher education and income, while ‘Western’ dietary patterns are more frequently followed by younger subject with lower incomes [31]. ‘Traditional’ patterns are more often reported in older subjects with lower education [33,34].

To the best of our knowledge, this is the first study to use FOP nutrition labels to characterize dietary behaviour using this type of methodology. Some studies have used FOP nutrition labels to investigate diet, but all considered a binary indicator of the nutritional quality of foods, which prevents direct comparisons across studies [35–38]. Lichtenstein et al. investigated the association between dietary behavior and consumption of American Heart Association (AHA) Heart Check Certifiable (HCC) foods [38]. Nutritional requirements for AHA HCC foods vary for 6 categories of foods, and include content in saturated fat and sodium (as in the 5-CNL) but also content in *trans*-fat, cholesterol, and some vitamins and minerals, leading to a binary assessment of the nutritional quality of the food (Heart Check vs. no Heart Check) [39]. Comparison with the 5-CNL suggests that AHA HCC foods would correspond to ‘Green’ labeled foods. In the NHANES population, consumption of AHA HCC foods was positively associated with consumption of fruit, vegetables and total grain, and inversely associated with the percentage of energy from saturated fat, alcohol, cholesterol and sodium [38]. These results are consistent with ours, as subjects with high consumption of AHA HCC foods would more likely be in the ‘Healthy diet’ cluster. Another study showed that the NuVal system, which ranks foods from 0 to 100 according to their content in 30 different nutrients, applied to individual diets was correlated to the Healthy Eating Index and to the DASH diet [37]. Such results are in line with ours, as subjects with high ONQI would more likely correspond to the ‘Healthy’ pattern. However, the NuVal system differs from the 5-CNL, as it delivers a quantitative continuous information to the consumer (0–100), compared to five classes of nutritional quality. Moreover, no direct comparison was possible with the FSA score, given that the NuVal uses a proprietary algorithm [40].

Our results are in line with modelling studies investigating the potential impact of substitutions within the diet [41–44]. Vyth et al. simulated the effect of substituting foods not complying with the Dutch *Choices* program with complying foods in the Dutch population [44]. In a maximal scenario where diets were simulated to include 100% of foods complying with the *Choices* program, 60.2% of the population complied with the recommended sodium intake, 54.0% to the recommended saturated fatty acids intake and 32.2% to the recommended sugar intake. In comparison, in scenario n°1 from our study, 91.0% to 91.7% of subjects (in the ‘Western’ and ‘Healthy’ patterns respectively) reached the recommended intake for sodium, 85.7% to 91.7% of subjects (same patterns) reached the recommended intakes for saturated fatty acids and 94.5% to 99.8% of subjects reached the recommended intakes for added sugars. Though the recommended amounts set at targets in the paper by Vyth et al. were more stringent than those we used, the observed differences suggest that the 5-CNL may have a higher impact compared to the Dutch Choices program. These differences can be partly explained by the fact that, contrarily to the Choices program, the 5-CNL would apply to all foods, allowing for a larger number of possible substitutions across the spectrum of available foods, including those with lower nutritional quality.

Modeling studies using observational dietary data have shown that both an increase in the panel of foods consumed and substitutions of foods would be required to achieve a balanced diet [45,46]. However, modifying the structure of one’s diet with the introduction of unfamiliar foods is constraining, while substitutions appear as more easily achievable. Therefore, at the individual level, the 5-CNL could be a helpful tool in implementing substitution strategies, allowing for an improvement in the nutritional quality of the diet. Subjects with already healthy diets would however have a lower gain from this strategy, while the potential lever for improvement in subjects with lower quality diets is more important. Indeed, the relative increase in the number of subjects reaching the recommended amount of saturates in the diet using scenario n°3 (the less constraining) is 11.7% in the ‘Healthy’ pattern compared to 32.4% in the ‘Western’ pattern. At the population level, even if subjects with lower quality diets would use the 5-CNL less, the gain in terms of nutritional quality would be more important. This aspect of the 5-CNL is to be taken into account while the major focus of public health nutrition in France is tackling social inequalities in health, including nutrition [47,48]. However, the introduction of a FOP nutrition label is only one of the multiple contributors that might help in improving the diet at the population level, along with other actions in the framework of a national program. The expectations in terms of impact for the 5-CNL should therefore take into account its connection and consistency with education, information and regulation policies.

Two methodological elements concur to the magnitude of the simulated effect in a substitution scenario: the level of aggregation of foods (the higher the aggregation, the higher the effects) [43], and the number of foods substituted (the higher the number of foods substituted, the higher the effects) [42]. In our study, foods were regrouped using a moderate level of aggregation, and considering food groups with similar use. The number of foods substituted was also considered in the third scenario, where 30% of foods were eligible for substitution. However, the use of the 5-CNL in real-life settings might lead to other substitution strategies, which we were not able to take into account. Indeed, substitutions could occur for similar products from different brands rather than other foods within the same food group. The potential impact of such substitutions should also be evaluated, in order to better take into account all the strategies that could be implemented in real-life settings. Finally, it is probable that in real-life setting, only a fraction of the population will use the FOP nutrition label, to substitute also a fraction of the foods they consume. Future studies should therefore simulate the potential impact of the FOP system, taking into account not only the number of foods substituted, but also the number of subjects using the FOP system.

Strengths of our study include its large sample size, and the use of validated dietary collection data, using repeated dietary records [49]. Moreover, the implementation of several substitution scenarios allowed for a more detailed evaluation of the potential impact of the 5-CNL on the nutritional quality of the diet across dietary profiles.

Our study is subject to some limitations. First, the actual use of the FOP system once implemented can’t be anticipated, as food choices at the point of purchase are influenced by individual characteristics (socio-demographical, lifestyle and dietary data), but also by the characteristics of the food offer (price, brands and marketing). A nutritional label would therefore be only one in many cues the consumer faces when grocery shopping. Stimulating awareness towards the label in itself can be considered as a challenge. Therefore, the actual use of the FOP label is likely to be low [50]. The results of the simulation scenarios presented in this paper should therefore be interpreted as best case scenarios and potential nutritional gains that could be achieved when using the label for food substitutions. Second, the substitution strategies we modelled do not take into account potential dietary modification induced by substitutions themselves (e.g. substitution from a sweetened yogurt to a non-sweetened option could lead to a concurrent increased consumption in sugar). Third, the use of the 5-CNL to implement substitutions in a real-life setting is likely dependent on the dietary pattern, which was not taken into account in the substitution models. Indeed, subjects who are more concerned about their diet, and therefore more likely to have ‘Healthy’ dietary patterns, are also more likely to use a nutrition label when grocery shopping [50]. Finally, our sample consists of volunteer subjects included in a cohort study on nutrition, who are therefore more likely to have healthier behaviours. Moreover, the methodology used is highly data-driven, which might also impair generalizability of our results. However, the dietary profiles that were identified included groups with less healthy dietary behaviours (‘Western’ and ‘Traditional’), which represented the majority of our sample (69.9% of the total sample). Moreover, the profiles identified in our study are similar to other studies, allowing for meaningful international comparisons [32].

More research is needed to help anticipating the potential of FOP systems to modify choices at the point of purchase, among all the factors influencing food choices.

Use of the 5-CNL to identify food choices allowed for the identification of three meaningful dietary patterns. Moreover, the 5-CNL could be a useful tool to help consumers substitute foods for a healthier alternative, and reach significant nutritional improvements in the diet, especially in individuals with poorer diets.

## Supporting Information

S1 Fig Scenarios of substitution used in the simulations. (PPTX)

S1 Table Food groups used for substitution strategies. (DOCX)

S2 Table Cut-offs used to define adequate intakes in energy, macro- and micronutrients. (DOCX)

## Abbreviations

5-CNL: Five-colour nutrition label
DI: Dietary Index
FOP: Front-of-pack
FSA: Food Standards Agency
FSA-NPS DI: Food Standards Agency Nutrient Profiling System Dietary Index
NPS: Nutrient Profiling System
NuVal: ONQI score denomination
OfCom: Office of Communication
ONQI: Overall Nutritional Quality Index
PNNS: Programme National Nutrition Santé
UK: United Kingdom
