# Supplementary material for: The 5-CNL Front-of-Pack Nutrition Label Appears an Effective Tool to Achieve Food Substitutions towards Healthier Diets across Dietary Profiles
Source: PLoS One. 2016 Jun 20;11(6):e0157545. doi: 10.1371/journal.pone.0157545 (PMC4913953; doi:10.1371/journal.pone.0157545)
Supplement: S1 Fig — (PPTX) [file pone.0157545.s001.pptx]

## Slide 1
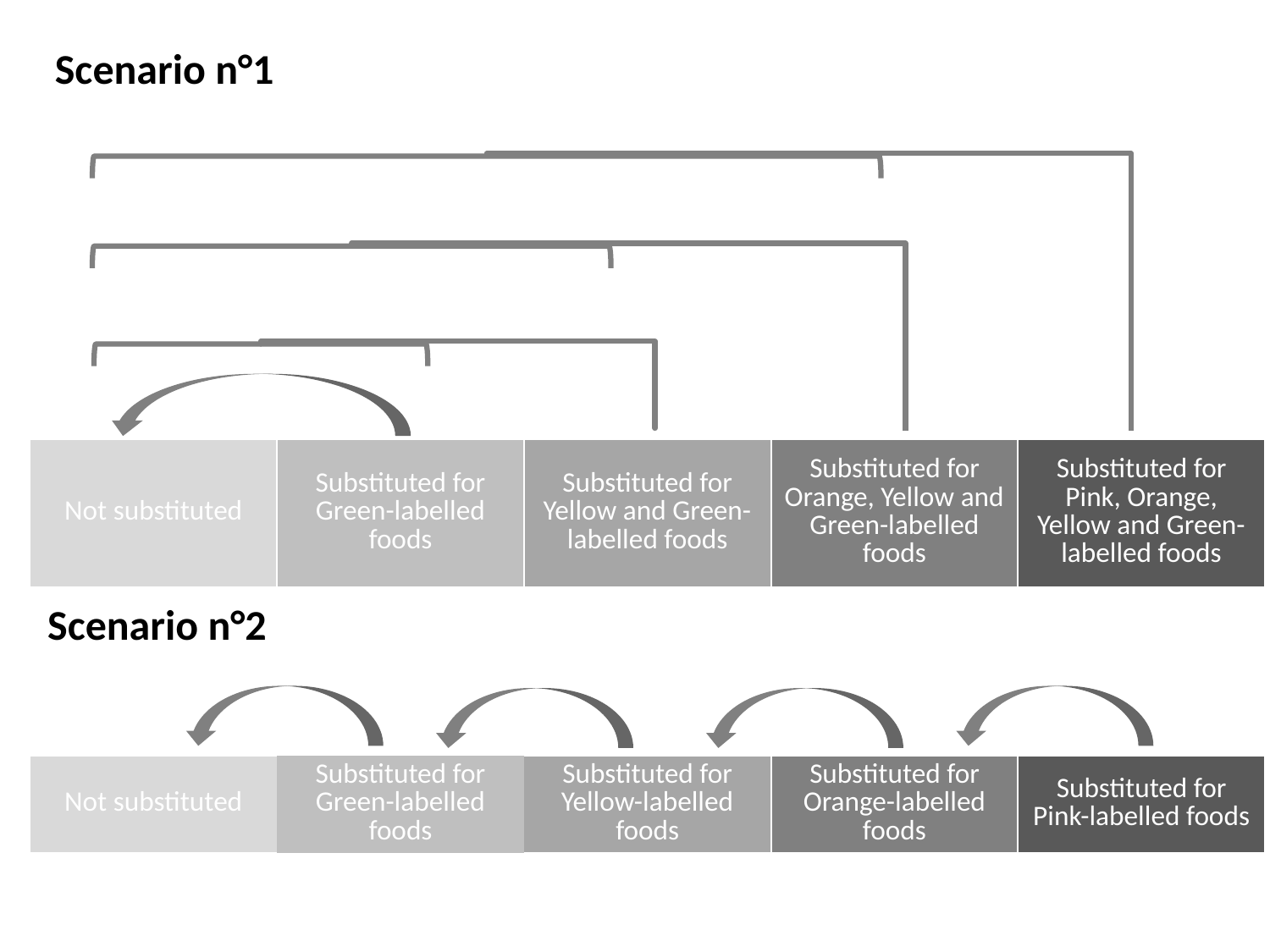

Scenario n°1
| Not substituted | Substituted for Green-labelled foods | Substituted for Yellow and Green-labelled foods | Substituted for Orange, Yellow and Green-labelled foods | Substituted for Pink, Orange, Yellow and Green-labelled foods |
| --- | --- | --- | --- | --- |
Scenario n°2
| Not substituted | Substituted for Green-labelled foods | Substituted for Yellow-labelled foods | Substituted for Orange-labelled foods | Substituted for Pink-labelled foods |
| --- | --- | --- | --- | --- |
