# Supplementary material for: The 5-CNL Front-of-Pack Nutrition Label Appears an Effective Tool to Achieve Food Substitutions towards Healthier Diets across Dietary Profiles
Source: PLoS One. 2016 Jun 20;11(6):e0157545. doi: 10.1371/journal.pone.0157545 (PMC4913953; doi:10.1371/journal.pone.0157545)
Supplement: S1 Table — (DOCX) [file pone.0157545.s002.docx]

Supplemental Table 2 Food groups used for substitution strategies

| **Beverages** |
| --- |
| Hot beverages |
| Cold beverages |
| Artificially sweetened beverages |
| Alcoholic-free beverages |
| Sweetened beverages |
| Fruit and vegetable juice |
| Fruit nectar |
| **Starchy foods** |
| Flour |
| Rusks |
| Whole grain cereals |
| Legumes |
| Bread |
| Potatos |
| Non-sweetened breakfast cereals |
| Sweet breakfast cereals |
| Tubers |
| **Fruit and vegetables** |
| Broth |
| Raw fruits |
| Cooked fruits |
| Dried fruits |
| Raw vegetables |
| Cooked vegetables |
| Vegetable soups |
| **Fats and sauces** |
| Vegetable oils |
| Hot sauces |
| Cold sauces |
| Butter and margarines |
| Salad dressings |
| **One-dish meals** |
| Pizza, pie and quiche |
| Fish one-dish meal |
| Meat one-dish meal |
| Sandwich |
| Vegetarian one-dish meal |
| **Dairy products and fresh desserts** |
| Dairy desserts and fresh desserst |
| Cheese |
| Cottage cheese |
| Ice cream |
| Milk |
| Yogurt |
| **Salty snacks** |
| Nuts |
| Aperitives |
| **Sugary snacks** |
| Cereal bars |
| Chocolate bars |
| Biscuits |
| Confectionery |
| Cakes |
| Ready to fry |
| Pastries |
| Marmelade, honey |
| Chocolate |
| Pâtisserie |
| **Meat, fish and eggs** |
| Offals |
| Seafood |
| Ham |
| Shellfish |
| Eggs |
| Fish |
| Processed meat |
| Meat |
| Poultry |
