# Supplementary material for: The 5-CNL Front-of-Pack Nutrition Label Appears an Effective Tool to Achieve Food Substitutions towards Healthier Diets across Dietary Profiles
Source: PLoS One. 2016 Jun 20;11(6):e0157545. doi: 10.1371/journal.pone.0157545 (PMC4913953; doi:10.1371/journal.pone.0157545)
Supplement: S2 Table — (DOCX) [file pone.0157545.s003.docx]

**Supplemental Table 2 cut-offs used to define adequate intakes in energy, macro- and micronutrients.**

| Nutrient | Cut-off for adequate intake |
| --- | --- |
| Energy intake | <105% expenditures (1) |
| Carbohydrates | >50% of energy intakes (alcohol excluded)(2) |
| Added Sugar | <12.5% of energy intakes (alcohol excluded)(2) |
| Lipids | <36.5% of energy intakes (alcohol excluded)(2) |
| Saturates | <35% of lipid intakes(2) |
| Proteins | <2.2 g/kg (3) |
| Fibers | >25g/day(2) |
| Sodium | <2560 mg/day in women  <3150mg/day in men (2) |

Reference List

1. Estaquio C, Kesse-Guyot E, Deschamps V, Bertrais S, Dauchet L, Galan P, Hercberg S, Castetbon K. Adherence to the French Programme National Nutrition Sante Guideline Score Is Associated with Better Nutrient Intake and Nutritional Status. J Am Diet Assoc 2009;109:1031-41

2. Ministère du travail, de l'emploi et de la santé. Programme National Nutrition Santé 2011-2015. 2011. Paris, Ministère du travail, de l'emploi et de la santé. 10-9-2015.

3. AFSSA. Apports en protéines: consommation, qualité, besoins et recommandations. 2007. Paris, Agence Française de Sécurité Sanitaire des Aliments. 14-9-2015.
